# Risk factors for cutaneous myiasis (blowfly strike) in pet rabbits in Great Britain based on text-mining veterinary electronic health records

**DOI:** 10.1016/j.prevetmed.2018.03.011

**Published:** 2018-05-01

**Authors:** Rachel Turner, Elena Arsevska, Beth Brant, David A. Singleton, Jenny Newman, PJ-M Noble, Philip H. Jones, Alan D. Radford

**Affiliations:** a Institute of Veterinary Science, University of Liverpool, Leahurst Campus, Chester High Road, Neston, CH64 7TE, UK; b Institute of Infection and Global Health, University of Liverpool, Leahurst Campus, Chester High Road, Neston, CH64 7TE, UK

**Keywords:** Blowfly strike, Myiasis, Rabbits, Risk factors, Seasonality, Electronic health records

## Abstract

Blowfly strike is a devastating and often rapidly fatal disease in rabbits. In Great Britain (GB), *Lucilia sericata* is the primary causative species. Despite its severity, there has been minimal investigatory work into the disease in rabbits. Here we used text mining to screen electronic health records (EHRs) from a large sentinel network of 389 veterinary practices in GB between March 2014 and April 2017 for confirmed cases of blowfly strike in rabbits.

Blowfly strike was identified in 243 of 42,226 rabbit consultations (0.6%), affecting 205 individual rabbits. The anatomical site of recorded blowfly strike lesions was overwhelmingly the perineal area (n = 109, 52.4%). Less commonly lesions were observed affecting other areas of the body (n = 9, 4.3%) and head (n = 8, 3.8%); in 83 consultations (39.9%), the affected area was not specified. Of the rabbits presenting with blowfly strike, 44.7% were recorded as being euthanized or died.

A case control study was used to identify risk factors for blowfly strike in this population. Whilst sex and neuter status in isolation were not significantly associated with blowfly strike, entire female rabbits showed a 3.3 times greater odds of being a case than neutered female rabbits. Rabbits five years of age and over were more than 3.8 times likely to present for blowfly strike. For every 1 °C rise in environmental temperature between 4.67 °C and 17.68 °C, there was a 33% increase risk of blowfly strike, with cases peaking in July or August. Overall blowfly strike cases started earlier and peaked higher in the south of Great Britain. The most northerly latitude studied was at lower risk of blowfly strike than the most southerly (OR = 0.50, p < 0.001). There appeared to be no significant relationship between blowfly strike in rabbits and either the sheep density or rural and urban land coverage types.

The results presented here can be used for targeted health messaging to reduce the impact of this deadly disease for rabbits. We propose that real-time temporal and spatial surveillance of the rabbit disease may also help inform sheep control, where the seasonal profile is very similar, and where routine surveillance data is also not available. Our results highlight the value of sentinel databases based on EHRs for research and surveillance.

## Highlights

• Flystrike was recorded in 0.6% of rabbit consultations collected over three years from 389 sentinel practices across the UK.
• Fortyfive percent of consultations resulted in the euthanasia or death of the animal.
• Rabbits five years of age and over were more than 3.8 times likely to present for blowfly strike.
• Female entire rabbits were at greatest risk, being 3.3 times more likely to be affected than neutered females.
• For every 1 °C rise in predicted environmental temperature, there was a 33% increase in risk of flystrike.

## 1 Introduction

Cutaneous myiasis, or blowfly strike, occurs worldwide, and is caused by a variety of species of fly (Bisdorff and Wall, 2006; Hall, 1997). Rabbits, which are a frequently kept as companion animals in Great Britain (GB) (Sánchez-Vizcaíno et al., 2017), are commonly affected (Cousquer, 2006). Using a retrospective questionnaire study, it was shown that 94.5% of practices in England and Wales reported treating at least one case of blowfly strike between May and September 2005, with many affected rabbits dying (Bisdorff and Wall, 2006). Rabbits affected by blowfly strike typically develop tachypnoea, hypothermia, anaemia, considerable soft tissue damage, and if left untreated, can develop toxaemia, shock and rapid death (Ipek and Ipek, 2012). Overweight rabbits may be less able to clean themselves and thus more prone to blowfly strike (Cousquer, 2006). As such, blowfly strike is a notable welfare concern in affected animals.

The disease also heavily impacts on sheep, and is associated with a heavy economic burden to the industry, estimated at $280million dollars per annum in Australia (Sotiraki and Hall, 2012). In GB, 75% of farms had been struck with blowfly strike (Bisdorff et al., 2006), particularly in the south of England, with approximately 1.5% of sheep affected each year (French et al., 1995), reaching 12–15% on some farms in the absence of blowfly strike control (Broughan and Wall, 2007).

The parasite species most commonly associated with blowfly strike in GB is *Lucilia sericata,* the Common Green Bottle Fly (Hall and Wall, 1995), the life-cycle of which primarily drives the seasonality and incidence of disease. Data on the life cycle of *L. sericata* is generally available from work on sheep, although likely to be similar in rabbits. The predicted average minimum threshold temperature below which oviposition does not occur is approximately 8.5 °C (Broughan and Wall, 2007). Flies are attracted to susceptible areas by volatile substances produced by bacterial degradation of skin and wool (Ashworth and Wall, 1994). At permissive temperatures, an individual adult female fly can lay about 200 eggs on their vertebrate host, mainly on damp areas of the skin or coat (usually anal and perineal areas) (Sotiraki and Hall, 2012). Hatching takes approximately 24–48 h, and when they hatch, the larvae move down the fur toward the skin where they moult twice (Wall and Lovatt, 2015); this process takes about three days to complete. After feeding on the superficial epidermis, lymphatic exudates and necrotic tissue, the wandering third stage larvae drop from the host and enter the soil. In the autumn, they enter a winter diapause and only pupate and emerge the following spring.

Despite the severity of the disease, to date there has been little attempt to assess the risk factors for rabbit blowfly strike across GB. Furthermore, there is no compulsory surveillance for blowfly strike in GB in any species and thus the evaluation of disease burden is a challenge. Recently, electronic health records (EHRs), which can be collected at large temporal and spatial scales and in real-time from sentinel networks of veterinary practices, have been used to provide new insight into population health for a range of diseases and syndromes (Arsevska et al., 2017; O’ Neill et al., 2017), and including ticks (Sánchez-Vizcaíno et al., 2016; Tulloch et al., 2017). The aim of this study was to identify the prevalence, seasonality and risk factors of blowfly strike in companion domestic rabbits using veterinary EHRs available collected from the Small Animal Veterinary Surveillance Network (SAVSNET), a sentinel network of veterinary practices across GB (Sánchez-Vizcaíno et al., 2017).

## 2 Material and methods

### 2.1 Data extraction and inclusion criteria

Electronic health records (EHRs) were collected in near real-time through SAVSNET from volunteer veterinary practices across GB; a full description of the data collection protocol has been described elsewhere (Sánchez-Vizcaíno et al., 2017). Briefly veterinary practices using practice management software previously made compatible with SAVSNET participation and data exchange were recruited based on convenience. In participating practices, data is collected from each booked consultation (where an owner has booked an appointment to see a veterinary surgeon or nurse). Owners attending participating practices are given the option to opt out at the time of their consultation, thereby excluding their data. For those that participate, data are collected on a consultation-by-consultation basis and include information about the animal (e.g. species, breed, sex, neuter status, age, owner’s postcode, insurance and microchipping status), as well as free-text clinical narrative (subsequently automatically redacted for inadvertent personal identifiers), treatments given, and the vaccination history (Sánchez-Vizcaíno et al., 2017). Data collection and use by SAVSNET is ethically approved by the University of Liverpool Research Ethics Committee (RETH000964).

In order to explore the seasonality and risk factors for blowfly strike, we defined cases as those animals that were presented for consultation with active blowfly strike lesions, visually confirmed and recorded by the attending veterinary practitioner.

Clinical narratives that had been processed to remove any personal identifiers, were filtered to identify narratives referencing blowfly strike using regular expressions (regex; https://en.wikipedia.org/wiki/Regular_expression) to detect the word blowfly strike, identified term variants and misspellings. The final regex (Supplementary figure) when applied to the full SAVSNET rabbit database of 42,226 rabbit consultations identified 443 consultations. This data was manually read by two authors (RT and EA), identifying 243 consultations (54.8% of those extracted by the regex) that satisfied the case definition of active blowfly strike disease. Of these 243, any rabbits that had visited the veterinary practitioner more than once in a month were considered to be suffering from the same episode of blowfly strike and thus only the first consultation was kept. Furthermore, rabbits with incomplete data (e.g. age or owner postcode not recorded) were also excluded from analyses. The final dataset consisted of 205 rabbits presenting with blowfly strike; three of these rabbits had two separate blowfly strike episodes longer than one month apart giving a total of 208 episodes of blowfly strike (Fig. 2).

In order to determine risk factors associated with blowfly strike, we conducted a retrospective case-control study; control rabbit consultations (979, 1 case: 4 controls) were randomly chosen from those rabbits that had never presented for an episode of flystrike (based on the clinical narratives of the electronic health records).

### 2.2 Animal data

For each case and control, the unique consultation ID was used to extract the rest of the information for that animal, including the postcode of the owner, the breed, sex, neuter status and date of consultation. Age was calculated as the difference in years between the date of birth and the date of consultation of each animal, and was categorised into quintiles (Table 1). Manual reading of clinical narratives was also used to classify if recorded the site of blowfly strike lesions, body condition score, as well as any other relevant clinical or outcome data. Information on the housing of the rabbit was not routinely available.

**Table 1:** Univariable logistic regression investigating ten variables as potential predictors of blowfly strike cases in rabbits from 389 veterinary practices throughout GB.

Variable	Level	Case (%)	Control (%)	Beta	OR (95%CI)	p-value
Sex	Male (Intercept)	101 (15.7)	541 (84.3)	−1.68	0.19 (0.15–0.23)	0.00
	Female	107 (19.7)	437 (80.3)	0.27	1.31 (0.97–1.77)	0.07

Neuter status	Neutered (Intercept)	78 (14.4)	463 (85.6)	−1.78	0.19 (0.13–0.21)	0.00
	Entire	130 (20.2)	515 (79.8)	0.40	1.50 (1.1–2.03)	0.73

Sex and neuter status	Female neutered (Intercept)	24 (11.5)	184 (88.5)	−2.04	0.13 (0.08–0.2)	0.00
	Female entire	83 (24.7)	253 (75.3)	0.92	2.51 (1.54–4.10)	<0.001
	Male entire	47 (15.2)	262 (84.8)	0.32	1.38 (0.81–2.33)	0.23
	Male neutered	54 (16.2)	279 (83.8)	0.39	1.4 (0.89–2.49)	0.13

Age	0–1 years (Intercept)	12 (11.4)	105 (89.7)	−2.17	0.11 (0.06–0.21)	0.00
	1–3 years	48 (21.1)	227 (82.5)	0.61	1.85 (0.94–3.63)	0.07
	3–5 years	50 (26.0)	192 (79.3)	0.82	2.28 (1.16–4.47)	0.02
	5–7 years	52 (42.6)	122 (70.1)	1.31	3.73 (1.89–7.36)	<0.001
	>7 years	36 (48.6)	74 (67.3)	1.44	4.26 (2.08–8.73)	<0.001

Urbanisation	Rural (Intercept)	51 (16.0)	268 (84.0)	−1.66	0.19 (0.14–0.26)	0.00
	Urban	157 (18.1)	710 (81.9)	0.15	1.16 (0.82–1.64)	0.39

Season	Summer (Intercept)	145 (34.9)	270 (65.1)	−0.62	0.54 (0.44–0.66)	0.00
	Spring	26 (9.3)	253 (90.7)	−1.65	0.19 (0.12–0.30)	<0.001
	Autumn	34 (12.5)	239 (87.5)	−1.33	0.26 (0.17–0.40)	<0.001
	Winter	3 (1.4)	216 (98.6)	−3.65	0.03 (0.01–0.08)	<0.001

Latitude	Lat1 (50.3–51.3] (Intercept)	50 (21.0)	188 (79.0)	−1.32	0.26 (0.19–0.36)	0.00
	Lat2 (51.3–51.8]	47 (19.8)	190 (80.2)	−0.07	0.93 (0.60–1.45)	0.75
	Lat3 (51.8–52.7]	40 (16.9)	197 (83.1)	−0.27	0.76 (0.48–1.21)	0.25
	Lat4 (52.7–53.6]	43 (18.1)	194 (81.9)	−0.18	0.83 (0.53–1.31)	0.43
	Lat5 (53.6–57.7]	28 (11.8)	209 (88.2)	−0.68	0.50 (0.30–0.83)	<0.001

Average monthly temperature	(Intercept)	–	–	−5.15	00.0	0.00
	Continuous			0.29	1.34 (1.27–1.40)	<0.001

Average monthly precipitation	(Intercept)	–	–	−0.36	0.7 (0.38–1.3)	0.24
	Continuous			−0.02	0.98 (0.97–0.99)	<0.001

Sheep density	(Intercept)	–	–	−1.50	0.22 (0.18–0.26)	0.00
	Continuous			−0.00	0.99 (0.99–1.00)	0.31

OR, odds ratio, CI, confidence interval.

The SAVSNET database also collects the breed of the animals. Some breeds have longer coats or more dense fur, which may predispose them to faecal soiling (Cousquer, 2006). However, of the 208 cases of blowfly strike identified, 140 had missing information about the breed, and thus the breed of the rabbit was not analysed.

### 2.3 Climate data and seasonality

The seasonality of blowfly strike was evaluated by meteorological season: winter (December to February), spring (March-May), summer (June-August) and autumn (September to November). The monthly average (night and daytime) temperature (°C) and precipitation (mm) were assessed, to further quantify the influence of climate on the occurrence of blowfly strike (Fick and Hijmans, 2017). For each owner postcode and month of consultation, we extracted the average monthly temperature and precipitation using the 2.5 min spatial resolution rasters freely available from the WorldClim version 2 at: http://worldclim.org/version2 (Fick and Hijmans, 2017).

Furthermore, the spatial risk of blowfly strike was evaluated based on the coordinates (latitude, longitude) of owner postcodes. Latitudes were divided into five equal quintiles, each containing 20% of the cases, with Lat1 being the most southerly quintile. For each quintile the number of blowfly strike consultations per one thousand total rabbit consultations was calculated for each month.

Data on urbanisation was obtained from the 30 s spatial resolution raster of the dominant aggregate class version of the Land Cover Map 2015 available from the Centre for Ecology and Hydrology at: https://www.ceh.ac.uk/services/land-cover-map-2015. More precisely, the dominant cover of GB is divided into ten classes, i.e. 1 = broadleaf woodland, 2 = coniferous woodland, 3 = arable, 4 = improved grassland, 5 = semi-natural grassland, 6 = mountain, heath, bog, 7 = saltwater, 8 = freshwater, 9 = coastal, 10 = built-up areas and gardens (Rowland et al., 2017). For each point that represents the owner’s postcode, the characteristics of the land cover class were retrieved. The effect of urbanization on the occurrence of flystrike in rabbits was evaluated by dividing the cases occurred in built-up areas and gardens (urban areas) and all other land cover classes (rural areas).

In order to explore possible correlations between blowfly flystrike in rabbits and sheep population density, we extracted the predicted sheep population density for each owner postcode, using a 2.5 min spatial resolution raster, freely available from the Food and Agriculture Organization (FAO) at: http://www.fao.org/ag/againfo/resources/en/glw/GLW_dens.html.

### 2.4 Statistical analysis

Descriptive statistics were used to characterise key demographic variables of the blowfly strike-visiting rabbit population. Univariable logistic regression was conducted utilising case control status as a binary outcome variable. Every explanatory variable was explored, with a likelihood ratio test (LRT chi-squared test) being used to assess fit compared to a null model. Explanatory variables with an LRT of p-values ≤0.2 were included in an initial multivariable logistic regression model. Interactions between sex and neuter status were included in the initial multivariable logistic regression model. A backwards explanatory variable selection process was utilised in order to produce a model fit with the lowest Akaike information criterion (AIC) possible, with explanatory variables being removed if their inclusion resulted in an increase in AIC, and by allowing the linear model to be related to the response variable via its ‘logit’ link function. The following assumptions were made: response variable was binary, explanatory variables were linearly related to the log odds, explanatory variables were independent and there was no multicollinearity among the independent variables. All assumptions were checked and verified before analysis. Regression results were considered significant for p-values ≤0.05. All analyses were carried out using R language (version 3.2.0) (R Core Team, 2015). Spatial data were projected according to the British National Grid coordinate system, EPSG 27,700 and visualised using Q-GIS (version 2.18.9).

## 3 Results

**Fig. 1:**
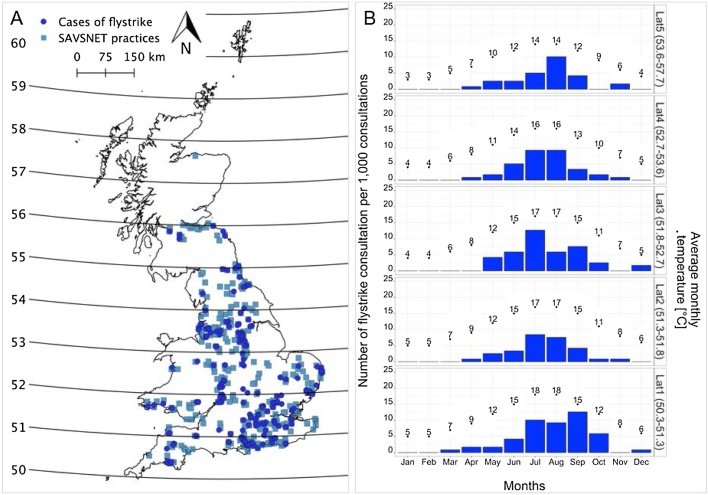
A: Distribution of blowfly strike cases across GB according to the longitude and latitude of the owner’s postcode. B: Percentage of blowfly strike cases in each month in each latitudinal quintile (Lat1 (50.3–51.3 degrees), Lat2 (51.3 to 51.8), Lat3 (51.8 to 52.7 degrees), Lat4 (52.7 to 53.6 degrees) and Lat5 (53.6 to 57.7 degrees)) and the average temperature per month in each quintile.

A total of 42,226 EHRs were collected between 25th March 2014 and 29th April 2017, from a total of 389 SAVSNET-participating veterinary practices throughout GB; the distribution of these practices is shown in Fig. 1. From these, 243 consultations (0.6%) were associated with flystrike, in 208 flystrike episodes involving 205 individual rabbits.

**Fig. 2:**
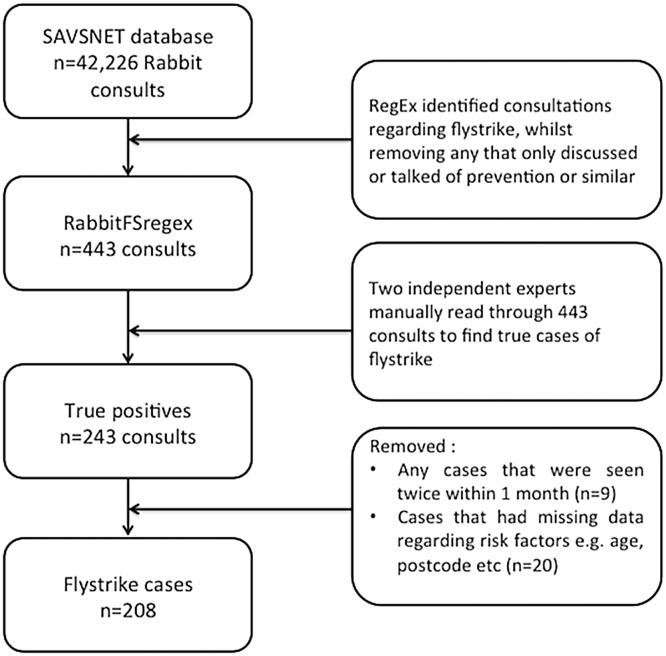
Inclusion and exclusion criteria outlining the process of extracting the true consultations and episodes of blowfly strike from the SAVSNET database.

The anatomical site of recorded blowfly strike lesions was overwhelmingly the perineal area (n = 109, 52.4%), including tail base, perineal, anal, vulva, penis and scrotum. Of these animals, 33 recorded either feacal soiling or urine scalding and 10 noted the rabbit suffering from paralysis or lameness of the hind legs. Those consultations that recorded lesions on the head (n = 8, 3.8%) were always accompanied with recorded ocular and/or nasal discharge. There were nine cases (4.3%) that were on non-perineal areas of the body such as the abdomen (n = 3) and flank (n = 6). In 83 (39.9%) consultations, the affected area was not specified. Additionally, among the affected rabbits, 13 were described as overweight (6.25%).

Of the 208 consultations categorised with blowfly strike, 92 recorded that the animal was euthanised and in one, the affected animal was dead on arrival giving a total of 44.7% of blowfly strike consultations leading to the death of the animal.

### 3.1 Univariable analysis

#### 3.1.1 Animal data

**Fig. 3:**
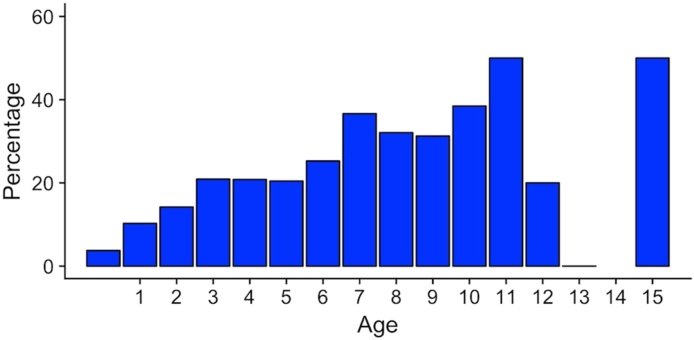
Percentage of rabbits at each age presenting with blowfly strike in the case control population.

The age profile of affected rabbits is shown in Fig. 3. An increase in risk of flystrike was as rabbits aged, and this became significant (p < 0.001) for rabbits aged 5–7 years (odds ratio (OR) = 3.73; CI = 1.89-7.36) and over seven years (OR = 4.26; CI 2.08–8.73) (Table 1). Neither a rabbit’s sex or neuter status were individually associated with flystrike risk. However, the inclusion of a gender and neuter status interaction term significantly improved the fit of the model, with entire females showing a 2.51 times higher risk (CI 1.54 – 4.10; p < 0.01) than neutered females.

#### 3.1.2 Climate data and seasonality

**Fig. 4:**
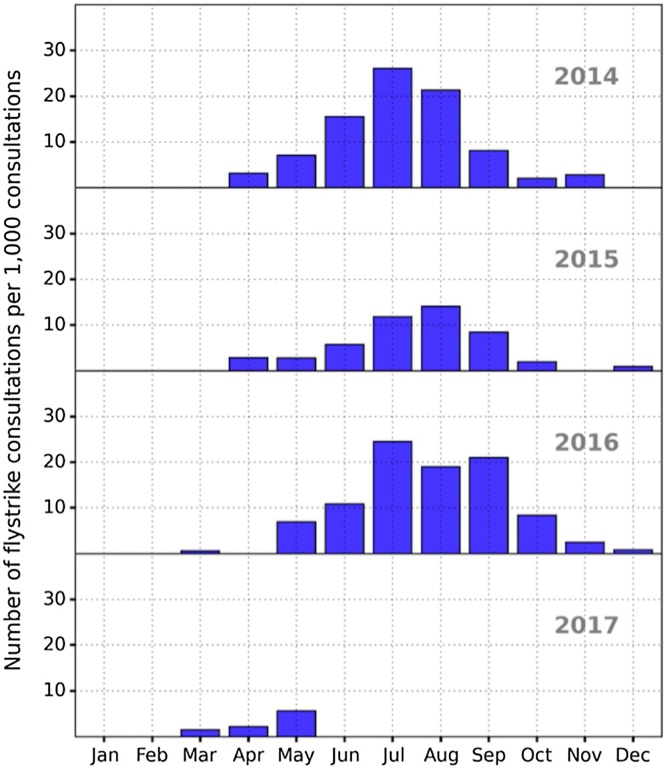
Annual seasonal pattern of blowfly strike consultations (per 1000 rabbit consultations) between April 2014 and May 2017.

As shown in Fig. 1, Fig. 4 there was a strong seasonal pattern in rabbit blowfly strike. The blowfly strike peak occurred in July in 2014 and 2016, and August in 2015. In 2014 and 2015, the first cases were identified in April, whereas in 2016 and 2017, the first cases were identified in March (Fig. 4). The majority of the cases (69.7%) occurred in summer (June-August). However, there were cases year-round in spring, autumn and winter (12.5%, 16.3% and 1.4% respectively). There was a significantly lower chance of blowfly strike in spring (OR = 0.19, p < 0.01), autumn (OR = 0.26, p < 0.01) and winter (OR = 0.03, p < 0.01) compared to summer (Table 1). Of the three cases recorded in winter, on reading the consultation, affected rabbits were described as very ill and weak, with a range of other health problems including excessive amounts of faecal soiling. There were no recorded cases in January and February.

The recorded episodes of blowfly strike consultations were in England (n = 194, 93.3%), Wales (n = 9, 4.3%) and Scotland (n = 5, 2.4%) (Fig. 1A).

The seasonality by latitude is shown in Fig. 1B. The earliest episode was recorded in latitude 1 (25th March). The latest episodes were in December in latitude 1 (n = 1) and 3 (n = 2). In each latitude, the profile of cases broadly followed the temperature, peaking at the same time as temperature with the exception of latitude 1, with cases peaking in September and temperature peaking in July and August.

Compared to latitude 1, the risk of blowfly strike was only significantly different (lower) for latitude 5 (OR = 0.5; CI 0.30–0.83; p < 0.001) (Table 1). For every 1 °C rise in temperature, there was a 34% increase in risk of blowfly strike (OR = 1.34; CI 1.27–1.40; p < 0.001) (Table 1). In addition, increased precipitation was associated with a small significant reduction in blowfly strike risk (OR = 0.98; CI 0.97–0.99; p < 0.001).

Urbanisation and sheep density had no significant association with blowfly strike risk (Table 1).

### 3.2 Multivariable analysis

Multivariable logistic regression showed the significant predictors of blowfly strike in this population were the rabbit’s age, the neuter status of female rabbits and the average monthly temperature (Table 2, Tables S1-3 from Supplementary Information).

**Table 2:** Finalised multivariable logistic regression model with the significant (p-values ≤0.05) variables predictors of blowfly strike cases in rabbits from 389 veterinary practices throughout GB.

Variable	Level	Beta	OR (95%CI)	p-value
(Intercept)	–	−6.42	0.00 (0.00–0.01)	0.00

Sex and neuter status	Female entire	1.19	3.30 (1.86–5.86)	<0.001
	Male entire	0.54	1.71 (0.93–3.15)	0.08
	Male neutered	0.58	1.79 (0.99–3.21)	0.05

Age	1–3 years	0.69	2.00 (0.97–4.11)	0.06
	3–5 years	0.81	2.25 (1.10–4.63)	0.03
	5–7 years	1.35	3.85 (1.85–S.01)	<0.001
	>7 years	1.56	4.78 (2.18–10.47)	<0.001

Average monthly temperature	Continuous	0.29	1.33 (1.26–1.40)	<0.001
AIC: 775.12				

OR, odds ratio; CI, confidence interval.

Rabbits age aged 5–7 were 3.85 times more likely to present for blowfly strike than young rabbits aged one year or less (CI 1.8–8.01; p < 0.001). This increased to 4.78 times for rabbits aged over seven years (CI 2.18–10.47; p < 0.001). Compared to female neutered rabbits, female entire rabbits showed a 3.30 greater risk (CI 1.86–5.86; p < 0.001); male entire and male neutered rabbits were not associated with altered risk to neutered does.

Analysis of temperature showed that, on average, each 1 °C increase in average monthly environmental temperature (4.67 °C–17.68 °C) resulted in a 33% increase in odds of blowfly strike (OR = 1.33; CI 1.26–1.40; p < 0.001). Only 11 consultations from the 208 were reported at times when the average monthly environmental temperature was below 8.5 °C, including one in March, two in April, and seven in November and December.

## 4 Discussion

Blowfly strike is recognised as an important welfare concern in both rabbits and sheep. However, the historical absence of routinely collected population health data for companion animal species, has led to an overall lack of understanding of important risk factors for the disease in rabbits. Here we have taken a novel approach to fill this gap using clinical narratives from EHRs available at large scale, confirming blowfly strike to be important in this population (0.6% of consultations of the SAVSNET network in GB), and identifying key risk factors that could inform improved targeted health messaging and control strategies.

Among the animals identified with confirmed blowfly strike in this population, 44.7% of cases ended in euthanasia or the death of the animal, much higher than the 2.7% reported for sheep (French et al., 1995). Blowfly strike is completely preventable and treatable if identified early (Cousquer, 2006), and such a high rabbit mortality reported here may suggest a lack of blowfly strike awareness amongst rabbit owners. Consistent with previous observations in both rabbits and sheep, the vast majority of blowfly strike affected the perineal area, where the greatest amount of feacal soiling and other organic matter which can attract flies may accumulate (Ashworth and Wall, 1994; Cousquer, 2006). This observation is also similar to sheep where 67–100% of blowfly strike cases affected the perineal area (Broughan and Wall, 2007; French et al., 1995). Other potential predisposing factors recorded concurrently in the rabbit EHRs included ocular discharge, paralysis, reduced grooming and overall poor coat hygiene.

The age of a rabbit was shown to have a significant association with the risk of blowfly strike in this population, with those aged five years and over being greater than 3.8 times more likely to present for blowfly strike than rabbits less than one year old. Older rabbits are likely to suffer more from lumbar spondylosis (reducing ability to bend and groom the perineal area), dental problems, decreased caecotrophy and less activity, and as a consequence, are less able to efficiently groom themselves (Cousquer, 2006). It is also possible that as some rabbits age, they receive less human interaction, such that owners may fail to, or take longer to, identify those health risks that predispose to blowfly strike.

Sex and neuter status were not independently associated with blowfly strike risk. However, when combined, entire female rabbits were 3.30 times more likely to suffer blowfly strike than neutered females. Currently we can only speculate on the causes underlying this observation. The rabbit breeding season occurs simultaneously with the main blowfly strike season in GB and female rabbits (does) can have multiple litters in one season (Boyd and Myhill, 1987; Cousquer, 2006). However it is unlikely that many of the entire does in our largely pet population were used for breeding so parturition, and associated lactation, are unlikely to explain the observed increase in risk. In the absence of pregnancy, pseudo pregnancy can occur following unsuccessful mating, or by mounting by another doe, and some does that live alone may be able to self-induce ovulation, and exhibit recurrent pseudo pregnancies (Richardson, 2000). However, for both pseudo pregnancy, and in entire female rabbits that are not mated, vaginal discharges are not generally considered to be significant features and therefore also seem unlikely to explain the observed risk. Entire does are also prone to developing obesity and uterine conditions as they age, most notably uterine adenocarcinoma, and less commonly endometrial hyperplasia, endometritis and pyometra (Cousquer, 2006). Finally, neutered does may also be cared for differently by their owners. A recent study in GB suggested neutering of rabbits was less common than for cats and dogs, with less than half of rabbits being recorded as neutered (45.8%); neutering was also less common in female rabbits (40.3%) than male rabbits (50.0%) (Sánchez-Vizcaíno et al., 2017). Understanding which features of entire female rabbits and their care places them at heightened risk for blowfly strike will help identify targeted health interventions aimed at reducing this risk.

Unsurprisingly, average monthly environmental temperature showed a stronger association with blowfly strike than any other environmental or geographical variable examined, explaining the pronounced seasonality of blowfly strike in the study population, with the majority of cases occurring in summer when the average monthly temperature is highest. Warmer climate in southerly latitudes of GB likely explains the earliest cases being recorded in the most southerly quintile in May. The more southerly regions also appeared to have a longer recorded blowfly strike season, and a higher proportion of cases during their peak months.

The geographical and seasonal patterns observed here in rabbits are consistent with those observed for sheep. A retrospective farmer survey showed the proportion of farms with at least one case of blowfly strike was highest in south-west England and lowest in Scotland (Bisdorff et al., 2006). The first sheep cases were reported in May (MacLeod, 1943), and the last case seen was in November (French et al., 1995). These similarities suggest surveillance data of risk in rabbits could also be used to indicate spatiotemporal risk in sheep, augmenting existing control measures for ovine myiasis; the work presented here could provide the foundation for such surveillance, especially if coverage were widened and analyses undertaken in near-real time.

Some cases of rabbit blowfly strike were identified in May in northerly latitudes and in November and December, when average environmental temperatures were below the predicted threshold for oviposition of 8.5 °C (Broughan and Wall, 2007). These cases may be associated with occasional periods where an environmental temperature of 8.5 °C is reached. However, it is also known that local factors within a sheep’s coat can provide the necessary micro-environmental conditions on the host itself at otherwise non-permissive environmental temperatures. A study of *Lucilia cuprina* (a relative of *L. sericata*) showed that flies can cause strike in sheep independent of temperature and high fly abundance, suggesting the availability of organic matter is a more important driver of blowfly strike development than environmental temperature for breach strike in some sheep (Wardhaugh et al., 2007). It is possible that the same may be true for rabbits, such that the humid environments of some hutches and damp bedding may extend the effective blowfly strike season.

Unlike temperature, precipitation was not deemed significant in the multivariable model, suggesting perhaps not surprisingly that all months have sufficient rainfall in GB to create the local humidity necessary for blowfly strike to occur (Wall et al., 2001). Lack of association between land cover type or sheep density, suggests population densities of flies are not dependent on these characteristics. Female flies will lay their eggs on most decaying organisms including dead wildlife species and food material and it is likely that the wide availability of such opportunities means there is little geographical restriction to risk.

Although EHRs can provide new insight into disease, their use has certain limitations. Despite having a highly efficient system for extracting cases of blowfly strike from the SAVSNET database, some true cases of blowfly strike were likely missed either because they were not recorded, or because they were recorded in a way not picked up by the regex used. As such the level of disease reported here is likely to be an underestimate of the true incidence of blowfly strike. A large proportion of consultations in the study population lacked recorded breed information. This level of missing data is much higher than seen for cats and dogs (Sánchez-Vizcaíno et al., 2017), and suggests veterinary surgeons may feel less familiar with rabbit breeds and less confident to commit such information to the EHR. As a result we were unable to assess the association of long-hair type on blowfly strike risk; in sheep there is a pre-shearing peak of blowfly strike cases supporting the hypothesis that the disease more commonly affects longer wool or fur (Wall et al., 2011). Furthermore, the data available is restricted to animals that presented to SAVSNET-participating practices, who themselves were recruited largely by convenience; the results presented here may not therefore be generalizable to the entire population of rabbits attending veterinary practices in GB. Areas of the country that lack cases should not be used to indicate lack of risk as these are also frequently the areas from which SAVSNET receives limited data. The relatively small number of cases identified here likely reflects both blowfly strike being a relatively rare disease, and rabbits presenting less frequently to veterinary practices than dogs and cats (Sánchez-Vizcaíno et al., 2017); with increased recruitment and time in SAVSNET, more data can be gathered to give an improved picture of the state of this important disease.

In conclusion, this is the first study to confirm a countrywide seasonality of blowfly strike in rabbits. We have found that the main predictive risk factors for blowfly strike are increased age, being an entire female rabbit and increased average monthly temperature. Our use of the SAVSNET data illustrates the utility of large databases of EHRs to investigate disease burden, especially in companion animals, which otherwise lack coordinated disease surveillance. The results presented here can be used to generate targeted health messages for risks of blowfly strike aimed at rabbit owners and veterinary practitioners.

## Author’s contribution

RT designed and planned the study, performed all preliminary data analyses and drafted the manuscript. EA, PJ and DS finalised the statistical and epidemiological analyses. AR conceived and helped design the study, and recruit practices, and wrote the final manuscript. AR, BB, PJ and PJN helped fund the project, collected the data and participated in the epidemiological analyses. All authors read, edited and approved the final manuscript.

## Competing interests

The authors declare that they have no competing interests

## Availability of data and materials

The datasets used and/or analysed during the current study are available from the corresponding author on reasonable request.
