# Supplementary material for: Risk factors for cutaneous myiasis (blowfly strike) in pet rabbits in Great Britain based on text-mining veterinary electronic health records
Source: Prev Vet Med. 2018 May 1;153:77–83. doi: 10.1016/j.prevetmed.2018.03.011 (PMC5910172; doi:10.1016/j.prevetmed.2018.03.011)
Supplement: Supplementary file 1 [file mmc1.docx]

**Supplementary tables**

Table S1. Initial multivariable logistic regression model investigating six variables as potential predictors of blowfly strike cases in rabbits from 389 veterinary practices throughout Great Britain.

| **Variable** | **Level** | **Beta** | **OR (95%CI)** | **p-value** |
| --- | --- | --- | --- | --- |
| **(Intercept)** | - | -4.44 | 0.01 (0.00-0.10) | 0.00 |
| **Sex and neuter status** | Female entire | 1.29 | 3.63 (2.02-6.51) | <0.001 |
|  | Male entire | 0.58 | 1.79 (0.97-3.32) | 0.06 |
|  | Male neutered | 0.62 | 1.85 (1.03-3.35) | 0.04 |
| **Age** | 1-3 years | 0.69 | 1.99 (0.96-4.12) | 0.06 |
|  | 3-5 years | 0.84 | 2.31 (1.11-4.79) | 0.02 |
|  | 5-7 years | 1.32 | 3.75 (1.78-7.89) | <0.001 |
|  | >7 years | 1.57 | 4.79 (2.17-10.61) | <0.001 |
| **Season** | Spring | -0.71 | 0.49 (0.25-0.95) | 0.03 |
|  | Autumn | -0.13 | 0.87 (0.45-1.71) | 0.69 |
|  | Winter | -1.44 | 0.24 (0.05-1.10) | 0.07 |
| **Latitude** | Lat2 (51.3 - 51.8] | -0.41 | 0.66 (0.38-1.16) | 0.15 |
|  | Lat3 (51.8 - 52.7] | -0.25 | 0.78 (0.45-1.37) | 0.39 |
|  | Lat4 (52.7 - 53.6] | -0.32 | 0.72 (0.42-1.26) | 0.25 |
|  | Lat5 (53.6 - 57.7] | -0.57 | 0.56 (0.30-1.04) | 0.07 |
| **Average monthly temperature** | - | 0.2 | 1.22 (1.11-1.35) | <0.001 |
| **Average monthly precipitation** | - | -0.01 | 0.99 (0.98-1.01 | 0.36 |
| **AIC: 780.93** |  |  |  |  |

OR, odds ratio; CI, confidence interval.

Table S2. Multivariable logistic regression model 2 investigating five variables as potential predictors of blowfly strike cases in rabbits from 389 veterinary practices throughout Great Britain.

| **Variable** | **Level** | **Beta** | **OR (95%CI)** | **p-value** |
| --- | --- | --- | --- | --- |
| **(Intercept)** | - | -4.99 | 0.01 (0.00-0.04) | 0.00 |
|  |  |  |  |  |
| **Sex and neuter status** | Female entire | 1.26 | 3.53 (1.98-6.32) | <0.001 |
|  | Male entire | 0.58 | 1.78 (0.96-3.30) | 0.07 |
|  | Male neutered | 0.61 | 1.84 (1.02-3.32) | 0.04 |
| **Age** | 1-3 years | 0.67 | 1.96 (0.95-4.05) | 0.07 |
|  | 3-5 years | 0.83 | 2.28 (1.10-4.73) | 0.03 |
|  | 5-7 years | 1.31 | 3.69 (1.75-7.76) | <0.001 |
|  | >7 years | 1.56 | 4.76 (2.15-10.54) | <0.001 |
| **Season** | Spring | -0.65 | 0.52 (0.28-0.99) | 0.05 |
|  | Autumn | -0.19 | 0.83 (0.43-1.60) | 0.58 |
|  | Winter | -1.38 | 0.25 (0.05-1.17) | 0.08 |
| **Latitude** | Lat2 (51.3 - 51.8] | -0.37 | 0.69 (0.40-1.19) | 0.18 |
|  | Lat3 (51.8 - 52.7] | -0.2 | 0.82 (0.47-1.42) | 0.47 |
|  | Lat4 (52.7 - 53.6] | -0.32 | 0.72 (0.42-1.26) | 0.25 |
|  | Lat5 (53.6 - 57.7] | -0.57 | 0.57 (0.31-1.05) | 0.07 |
| **Average monthly temperature** | - | 0.21 | 1.24 (1.12-1.36) | <0.001 |
| **AIC: 779.79** |  |  |  |  |

OR, odds ratio; CI, confidence interval.

Table S3. Multivariable logistic regression model 3 investigating four variables as potential predictors of blowfly strike cases in rabbits from 389 veterinary practices throughout Great Britain.

| **Variable** | **Level** | **Beta** | **OR (95%CI)** | **p-value** |
| --- | --- | --- | --- | --- |
| **(Intercept)** | - | -5.5 | 0.00 (0.00-0.02) | 0.00 |
|  |  |  |  |  |
| **Sex and neuter status** | Female entire | 1.2 | 3.32 (1.87-5.90) | <0.001 |
|  | Male entire | 0.55 | 1.74 (0.94-3.19) | 0.08 |
|  | Male neutered | 0.6 | 1.83 (1.02-3.28) | 0.04 |
| **Age** | 1-3 years | 0.66 | 1.94 (0.94-3.98) | 0.07 |
|  | 3-5 years | 0.78 | 2.19 (1.06-4.51) | 0.03 |
|  | 5-7 years | 1.31 | 3.71 (1.78-7.76) | <0.001 |
|  | >7 years | 1.54 | 4.68 (2.13-10.29) | <0.001 |
| **Season** | Spring | -0.56 | 0.57 (0.31-1.07) | 0.08 |
|  | Autumn | -0.07 | 0.93 (0.49-1.76) | 0.82 |
|  | Winter | -1.18 | 0.31 (0.07-1.40) | 0.13 |
| **Average monthly temperature** | - | 0.23 | 1.26 (1.15-1.38) | <0.001 |
| **AIC: 775.59** |  |  |  |  |

OR, odds ratio; CI, confidence interval.
