# Supplementary material for: Risk factors for cutaneous myiasis (blowfly strike) in pet rabbits in Great Britain based on text-mining veterinary electronic health records
Source: Prev Vet Med. 2018 May 1;153:77–83. doi: 10.1016/j.prevetmed.2018.03.011 (PMC5910172; doi:10.1016/j.prevetmed.2018.03.011)
Supplement: Supplementary file 2 [file mmc2.docx]

Supplementary figure 1

Final regular expression used to identify 443 consultations referring to flystrike / myiasis from a total of 42,226 rabbit consultations.

theFinalRegex = re.compile(r"""(?P<RabbitFS>

(?<!disc.)

(?<!cuss.)

(?<!sion.)

(?<!ssed.)

(?<!vise.)

(?<!rned.) # Removes any variant of discussed, advise, warned, prevent, risk, about, of, from

(?<!vent.)

(?<!risk.)

(?<!bout.)

(?<!..of.)

(?<!from.)

(?<!s/s/no.) # Removes no, and, re, for, avoid, diet, dietary, food, vaccination,

(?<!.and.)

(?<!..re.)

(?<!.for.)

(?<!void.)

(?<!iet..)

(?!<ing..) # Removes any variant identified in the output data that was a false positive

(?<!ant..)

(?<!dary..)

(?<!ood..)

(?<!are..)

(?<!vacc..)

(?<!.ion.)

(?<!ase..)

(?<!an..)

(?<!nce..)

(?<!rol..)

(?<!inst.)

(?<!ware.)

(?:Fly.*strike|mag+ot+|my?ia?sis)

(?!/scontrol)(?!/sprevent)(?!/sprotect)) #Removes flys strike that has been followed by control,

prevent, protect

""", flags=re.I|re.X)
